# A unique case of death by MDPHP with no other co-ingestion: a forensic toxicology case

**DOI:** 10.1007/s00414-022-02799-w

**Published:** 2022-02-15

**Authors:** Domenico Di Candia, Michele Boracchi, Barbara Ciprandi, Gaia Giordano, Riccardo Zoja

**Affiliations:** Dipartimento Di Scienze Biomediche Per La Salute-Sezione Di Medicina Legale E Delle Assicurazioni, Università Degli Studi Di Milano, via Luigi Mangiagalli, 37, 20133 Milan, Italy

**Keywords:** MDPHP, Synthetic cathinones, Autopsy, Lethal intoxication

## Abstract

Synthetic cathinones are a class of psychoactive drugs that have become, in recent years, of quite common observation in medical and toxicological forensic field. MDPHP (3,4-methylenedioxy-α-pyrrolidinohexanophenone) belongs to this class of substances but lethal acute intoxication caused by this specific substance without other co-ingestions has never been described in literature. We are presenting the unique case of a fatal acute intoxication caused by MDPHP in a 48-year-old man carried to the A&E department of a big Milanese hospital where he suddenly deceased after brief time. Clinical records, autopsy examination, histological findings, and toxicological analysis, assessed via a Q-Exactive Orbitrap with a HPLC system and LC/MS–MS system, are discussed. This case report may represent the first case of this type of intoxication and might help in improving recognition and treatment of these particular cases.

## Introduction

New psychoactive substances (NPS) are a class of recreational drugs with a wide range of pharmacological effects that have become, in recent years, a global phenomenon; this class of substances was originally designed as a legal alternative to those substances that were labeled as controlled molecules [1] and their use as “legal” recreational drugs is now well established [2]. These compounds are often purchased on the internet and are marketed as “bath salts,” “plant food,” or “research chemicals” [3]. Synthetic cathinones (SC) are a sub-group of NPS that possess hallucinogenic and psychostimulant properties and their action imitates the effects of other illegal substances such as cocaine, MDMA, and other amphetamines [4]. They are the among the most consumed drugs among NPS, can be much more potent than the natural products, and, in some cases, can be very dangerous and even have lethal effects [5].

MDPHP (3,4-methylenedioxy-α-pyrrolidinohexanophenone) is a molecule belonging to the sub-group of SC: this molecule is closely related to the more common MDPV (3,4-methylenedioxypyrovalerone) but literature about the lethal effects of this substance is very scarce.

According to literature, lethal intoxications caused by MDPHP are extremely rare and always combined with other SC: we are presenting the case of an acute intoxication caused by MDPHP without any other association and we are discussing clinical records, autopsy, histological, and toxicological data obtained.

## The case

At 8:30 a.m., a 48-year-old man was found semi-conscious on the sidewalk of a peripheric street of the city of Milan. The man was reporting an intense back and leg pain and the passers-by called for medical emergency support that arrived on the scene 3 min later. Questioned by the first responders, the patient confirmed the previous symptoms, said he was HIV + in therapy, and declared he had an amnesia preventing him from remembering anything happened in the past days until that moment. At first examination, the vitals were reported as follows: patient awake, regular breathing rate, peripheric circulation normal, heart rate rhythmic (73 bpm), blood pressure 110/80 mmHg, Sat O2 98%, breathing rate 16/min, skin of normal pinkish coloration. The man was carried to the closest hospital for further investigations and the general conditions reported an increase of heart rate (up to 110 bpm) and of breathing rate 20/min. A few minutes after hospitalization the patient became suddenly unconscious: medical records report no breathing, no circulation, pale skin, and asystole rate. Cardiopulmonary resuscitation was attempted for 30 min but despite all the clinical efforts, heart rate and consciousness were never restored and death was declared at 09:20 a.m..

Personal effects were seized by Police Officers while the corpse was carried to the Institute of Legal Medicine of the University of Milan for forensic investigations and kept in a refrigerated cell. Police Officers found 3 plastic envelopes in the pocket of the trousers containing a white powder with crystalline aspect. The Judicial Authority ordered autopsy, histological, and toxicological examinations 3 days after the death.

Relatives interviewed before autopsy examination stated that the man was missing from home since 4 days and that he was an occasional drug user.

## Material and methods

### Histological analysis of post-mortem specimens

Samples of tissues (brain, lungs, heart, liver, spleen, and kidneys), collected during autopsy examination, were fixated in 10% buffered formalin. After fixation, fragments of 2.0 × 0.5 cm were dehydrated using an Automatic Linear Tissue Processor (ATP700, Histo-Line Laboratories) in increasing ethanol solutions (80%, 90%, 95%, and absolute alcohol in three steps), were clarified in a xylene substitute (three steps), and then embedded in high fusion point paraffin (56–58 °C) in three steps. Two µ-thick slides were cut using a Reichert microtome from each sample. These slides were let drying overnight in oven at 37 °C. All sections were then stained with H&E staining and with Masson’s Trichrome staining according to Goldner. All the microscope slides were examined using a Leica DMR optic microscope and the most significant areas were photographed using a Leica DC300 F digital camera.

### Toxicological analysis of post-mortem specimens

All samples collected during autopsy examination — liquid matrices (femoral blood and urine), gastric content, and nasal swabs — were analyzed following the standard protocols of the Bureau of Legal Medicine of the University of Milan.

### Chemicals

All standards molecules involved in this study, Internal Standards (Proadifen hydrochloride SKF 525A) included, were purchased from Sigma-Aldrich. Solvents used in the extractive processes were purchased by Sigma-Aldrich (hydrochloric acid, methanol, and chloroform), VWR Chemicals (isopropanol, acetone, ethyl acetate, N-hexane, dichloromethane, and 2-methyl-2-propanol). Buffer solution pH 6.88 was purchased from PanReac AppliChem ITW Reagents.

### Specimen preparation and extraction

For Headspace HS-SPME analysis, 0.5 mL of femoral blood was placed in a headspace vial, was spiked with 0.5 mL of water at 0.1% of 2-methyl-2-propanol that was used as an internal standard, and hermetically sealed.

Biological samples of femoral blood, gastric content, and urine were prepared and extracted according to established standard sample procedures described in our previous work [6]. Femoral blood samples, urine, and gastric content were submitted to mixed-mode solid-phase extraction (Bond Elut Certify) after addition of 100 ng of internal standard (SKF 525-A Proadifen hydrochloride), dilution with buffer pH 6.88 (1:10 v/v) vortex mixing, and centrifugation. Acidic/neutral and basic fractions were separately eluted and then evaporated. After reconstitution with 100 µL of methanol, they were analyzed by reversed-phase HPLC coupled to positive ionization high-resolution MS (QExactive) operated in full-scan and MS/MS mode with different collision energies.

Nasal swabs were soaked in methanol and sonicated for 15’at 40 kHz. Methanol was then filtrated using 0.2-µm filters (Millipore) and 100 µL was placed in a conical bottom vial and analyzed via HPLC/MS–MS analysis.

### Instrumental conditions

Headspace HS-SPME analysis was assessed using a GC/MS (ThermoFisher Scientific, DSQII).

Full-scan analysis was assessed using a Q-Exactive Orbitrap with a HPLC system (Thermo Fisher Scientific, San Jose, CA, USA).

Confirmation and measuring of the substances were assessed using a HPLC/MS–MS Triple Quadrupole TSQ Fortis II (ThermoFisher Scientific, San Jose, CA). All instrumental conditions are already reported in another work from the same authors [6].

### Validation procedure

Q-Exactive Orbitrap with a HPLC system was calibrated in positive with a ThermoScientific Pierce LTQESI Positive Calibration Solution (Product Number 88322) and in negative with a ThermoScientific Pierce LTQESI Negative Calibration Solution (Product Number 88324).

Validation procedures were assessed according to the SWGTOX guidelines [7].

Bias% was calculated using 3 different samples of blank blood and 3 different samples of blank urine spiked with different concentrations of analyte (low 0.15 µg/mL; medium 0.8 µg/mL; high 1.6 µg/mL). Samples were analyzed over 5 runs and bias% was calculated to be ≤ 20% for both matrices. Precision was calculated concurrently with bias% and expressed as %CV resulting to be ≤ 10%.

Carryover was evaluated analyzing a blank sample after each specimen. Carryover could not be detected in any blank sample for any analyte present in specimens.

Calibration standards and quality controls (0.25, 0.5, 1.0, and 2.0 µg/mL) for HPLC/MS–MS analysis were prepared spiking 0.5 mL of blank whole blood samples with adequate amounts of MDPHP working solutions in a range 0.25–2.0 µg/mL. We built a six-point calibration curve (0.05–0.1–0.25–0.5–1.0 and 2.0 µg/mL) based on the peak area ratios of the analytes to the IS against nominal analyte concentration using a weighted 1/ × 2 linear regression. Calibration samples were analyzed over six runs. We tested the correlation over the whole range of concentrations to grant a linear regression; linearity was considered adequate when *r*^2^ ≥ 0.990 and CV % ≤ 10.0.

Carryover was evaluated as appropriate when no transitions of the analytes involved in the study could be detected in QC samples.

Sensitivity as in LOD (limit of detection) and LOQ (limit of quantification), precision, and accuracy were in accordance to the Scientific Working Group for Forensic Toxicology standard practices [7]. LOD was determined using the Decision Point Concentration method. Three samples of blank blood fortified at 0.01 µg/mL of analyte were analyzed over 3 runs. Detection and identification criteria were met in all runs. S/N for LOD was calculated with FreeStyle™ (ThermoScientific) on the peak area of the analyte/noise interference of the minor value obtained and was equal to 207. LOQ was defined as the lowest non-zero calibrator. Three samples of the lowest non-zero calibrator were analyzed over three runs and all the criteria as in detection, identification, bias, and precision were met. Analytes’ recovery was determined by comparing the mass spectrometric response of a set of MDPHP-free whole blood samples (6 samples) spiked with MDPHP before extraction at a final concentration of 0.05, 0.1, 0.25, 0.5, 1.0, and 2.0 µg/mL respectively and a second set of MDPHP-free samples (6 samples) fortified with analytes at the same final concentration after extraction. Absolute recovery (%) was determined by comparing the peak areas of the previous sets of samples. The matrix effect (%) was defined by comparing the areas of the peaks of a first set of extracted aqueous samples in the low, intermediate, and high concentration range (0.10, 0.850, and 1.6 µg/mL) and with the peak areas of a second set of MDPHP-free whole blood samples (3 sets of matrices pooled from 10 donors), fortified with MDPHP at low, medium, and high concentration after extraction. Values for matrix effect ranged between − 7.0 and + 12.0%. Inter-day and intra-day validations were assessed: intra-day validation was assessed using 5 replicates of spiked blood and urine samples in 1 day while inter-day was calculated on 5 spiked samples of blood and urine over 5 days, and accuracy and precision were calculated and found ≤ 20% in compliance with international guidelines.

In order to evaluate matrix interference, blank blood samples and urine samples from ten different sources were collected and analyzed demonstrating the absence of interferences.

## Results

Autopsy examination of the cadaver revealed some non-specific features: the brain resulted to be of normal size and weight (1110 g) but the parenchyma had a congest appearance and an homogeneous aspect. The examination of the thoracic area revealed several infiltrated bone fractures (sternal gladiolus at the III costal space and ribs from II to VI bilaterally on the middle-clavicle line). Lungs were of normal size and weight (approximately 660 g each) with regular coloration and scattered petechiae on the surface. Once cut, the parenchyma resulted to be congested and an abundant foamy liquid was noticed at squeezing. Heart examination showed an organ of regular aspect and weight (360 g) showing several hemorrhagic suffusion areas of the epicardium of the posterior wall of left atrium and ventricle. The hemorrhagic suffusion did not penetrate the endocardium. At cut, heart cavities and walls resulted to be regular in shape. Stomach inspection revealed an amount of gastric content measured at about 400 mL while bladder examination revealed an amount of urine quantifiable in a few mL. No other relevant findings were observed during examination.

The microscopic observation of the histological samples demonstrated a marked edema of the cerebral parenchyma, an extensive pulmonal edema with stasis, and a diffuse hemorrhagic infiltration of the epicardial posterior wall of the left atrium and ventricle.

Toxicological results were the following: as a first step, HPLC-HRMS full scan was applied to the cadaveric samples (femoral blood, urine, and gastric content) highlighting the presence of MDPHP in all matrices. The exact mass was calculated, confirming the investigated analyte (290.17507 m/z) matching with high probability on m/z Cloud Library search. Exact mass weight and characteristic ion fragmentation are reported in Table 1.

**Table 1:** Exact mass of the protonated molecule (precursor ion) and characteristic product ions of MDPHP along with the respective collision energies

Molecule	Precursor ion	Product ion	Collision energy (eV)
MDPHP	290.17507	219.1024	21
	290.17507	149.0241	27
	290.17507	140.1441	34

Following the standard working protocols of the Bureau of Legal Medicine of the University of Milan, confirmation of the analyte was assessed via a LC/MS–MS TSQ Fortis II Triple Quadrupole (ThermoFisher Scientific, San Jose, CA) via MS/MS mode. Femoral blood, urine, and gastric content were confirmed to be positive for MDPHP. The molecule of toxicological interest detected in the biological samples was measured using its fragment 219.10 m/z via MS/MS mode and results are reported in Table 2. Nasal swabs, however, resulted to be negative for any xenobiotic.

**Table 2:** Concentrations of the analyte in all matrices examined

Biological matrix	MDPHP (ng/mL)
Femoral blood	399
Urine	222
Gastric content	50
Nasal swabs	Negative

BAC (blood alcohol concentration) evaluated via Headspace HS-SPME analysis resulted to be of 0.42 g/L.

The three envelopes found by the police in the trousers of the victim resulted to have a net weight content of 0.387 g, 0.170 g, and 0.104 g. The content was analyzed by the Police Scientific Department via GC–MS analysis and confirmed the composition in MDPHP in all samples, and no other substances were detected.

## Discussion and conclusion

Synthetic cathinones were discovered in 2006 while searching for medications able to treat cocaine abuse and after their creation, they took their place among “legal” recreational drugs [2]. MDPHP (3,4-methylenedioxy-α-pyrrolidinohexanophenone, C_17_H_23_NO_3_—CAS 776994–64-0) is an α-pyrrolidinophenone with a structure similar to MDPV (3,4‐methylenedioxypyrovalerone) belonging to the group of SCs. MDPHP is a stimulant of the release of norephedrine and dopamine, and meanwhile inhibits the reuptake of these substances: the effects so created are characterized by a vigorous stimulation of the central nervous system (CNS) [3]: due to its pharmacological effects, this drug is usually administered with recreational purposes. According to literature, the most frequent signs and symptoms observed under the effects of SC are hyperthermia, tachycardia, severe psychosis, cardiac and neurological problems, and death [8].

Literature regarding the use of MDPHP is lacking solid records about the effect of this substance when taken singularly: a Swedish study reports concentrations and physical effects of this molecule on four subjects but all survived [2]. According to this latter study, serum concentrations of this SC ranged from < 1 to 14.3 ng/mL while urine concentrations were from 19 to 48 ng/mL. The Swedish publication highlighted several symptoms as in euphoria, panic attacks, aggressiveness, difficulties in breathing, and unpleasant feelings in the thorax region. Clinical observations included hypertension and hallucinations. Effects of this SC in association to other psychoactive substances are described in few cases [3, 9]. The work conducted by Grapp and colleagues reported 9 cases of poly-intoxication by MDPHP and other substances (drugs of abuse and prescription drugs). MDPHP concentrations in serum ranged from 3.3 to 140 ng/mL but according to the authors, the clinical findings (aggressive behavior, impaired balance, loss of consciousness, respiratory insufficiency, agitation, mental confusion, and suicidal thoughts) could not be ascribed to the use of MDPHP alone but rather as the result of poly-intoxication [3] and all patients survived. A study conducted by Kavanagh and colleagues highlighted the clinical record of two patients intoxicated by MDPH and α-PVP: both cases were screened for the presence of SC in urine. One subject reported suicidal thoughts, hypertension (180/130), and increased heart rate (83 bpm) while the other subject presented to the A&E agitated, anxious, and disoriented, with hypertension (150/80) and accelerated heart rate (91 bpm). Both subject had quite low BAC (0.35 and 0.39 g/L) [9]. Regarding the lethal effect of this substance, a single case of fetal death is described due to maternal administration of MDPHP and α-PHP (α-pyrrolidinohexiophenone): the mother started vomiting and was found unconscious in her apartment; when hospitalized, 2 h later, the woman showed psychomotor agitation, anxiety, and mumbled speech with hypertension (160/90) and increased heart rate (130 bpm). Fetal blood concentration of MDPHP was 76 ng/mL and α-PHP concentration was of 12 ng/mL while the mother’s blood concentration of MDPHP was 16 ng/mL and only traces of α-PHP could be determined in the hematic tissue [10]. The lethal effect, however, could not be ascribed solely to MDPHP but the association of two synthetic cathinones should be taken into consideration. Reports about the lethal effect of this molecule not in association with other substances are lacking completely.

All findings reported during autopsy examination were to be considered as non-specific: brain congestion, pulmonal petechiae, and lung congestion could be ascribed to *limine vitae* suffering while sternal and ribs infiltrated fractures and the hemorrhagic suffusion of the epicardium could be traced back to the prolonged cardiopulmonary resuscitation maneuvers that were sustained for 30 min.

Histopathological findings were in accordance with the macroscopic observation assessed during autopsy examination: cerebral edema, pulmonal edema, and hemorrhagic extravasation and infiltration observed in the epicardial context did not add any particular information to the case itself.

In our case report, the high concentration of MDPHP measured in femoral blood, corresponding to 399 ng/mL, could be considered as sufficient for causing the death of the subject. The concentration in the gastric content, equal to 50 ng/mL, the negativity of the analysis assessed on the nasal swabs, and the absence of injection marks on the body could trace back the administration modality to an oral ingestion of the substance. Considering the abundance of gastric content discovered at autopsy examination and the modest concentration measured of MDPHP at analysis, we can confidently state that most of the substance was already absorbed in the hematic circulation. The concentration of the substance in urine corresponded to 222 ng/mL: the quantity of urine contained in the bladder was of a few mL and we can not study the effective renal clearance of the substance. Urine collected during hospitalization was not preserved.

Considering the concentrations of the substance measured in different matrices and according to biological-statistical data, considering as well the witnessed period of time in which the subject was in the presence of passers-by or first responders, we feel confident in stating that the oral ingestion of the substance took place in a period of time included between 60 and 90 min before the arrival of the first responders. However, since literature regarding the solely ingestion of MDPHP is lacking, this data is a mere hypothesis and more specific studies should be carried out. The three envelopes sampled and analyzed by Police Officers of the Scientific Group of the Police by means of GC/MS analysis highlighting the presence of MDPHP lead to the reasoning that the administration was probably oral and voluntary. According to clinical records, the symptoms reported by the medical staff are not in discordance with a possible intoxication caused by SC: tachycardia, psychosis, and neurological problems are, in fact, frequently observed in these types of poisonings. The BAC value, corresponding to 0.42 g/L, can be considered as quite low and it is opinion of the authors that ethanol did not take part in the lethal effect of the SC. In conclusion, we can claim that death occurred because of acute intoxication cause by MDPHP with no other co-ingestion.

To authors’ knowledge, this case report is the first in literature reporting a death caused by acute intoxication from MDPHP without any other co-ingestion that could have played a role in the *exitus* of the patient. We can therefore affirm that this unique case could be considered as valuable in setting a first step for forensic evaluation of this type of deaths.
